# Effects of Psychological Resilience and Social Support on Anxiety and Depression in Patients With Papillary Thyroid Carcinoma

**DOI:** 10.62641/aep.v54i2.2181

**Published:** 2026-04-15

**Authors:** Yuan Ding, Ping Jiang, Ying Zhang

**Affiliations:** ^1^Department of Thyroid and Breast Surgery, South District of Hefei First People's Hospital, 233000 Hefei, Anhui, China

**Keywords:** papillary thyroid carcinoma, anxiety, depression, psychological resilience, social support

## Abstract

**Background:**

To investigate anxiety and depression in patients with papillary thyroid carcinoma (PTC) and analyse their associations with psychological resilience and social support.

**Methods:**

A total of 122 patients with PTC who received surgical treatment at our hospital between January 2022 and June 2023 were included. Clinical data of patients were collected. Psychological resilience, social support, and symptoms of anxiety and depression were evaluated using Connor–Davidson Resilience Scale (CD-RISC), Social Support Rating Scale (SSRS) and Hospital Anxiety and Depression Scale (HADS) at 1-month after surgery. Pearson correlation analysis was used to analyse the correlation among these indicators. And the influencing factors of postoperative anxiety and depression were analysed using multiple linear regression method.

**Results:**

All questionnaires were effectively collected. The total scores of CD-RISC, SSRS, and HADS were 80.00 ± 7.41, 53.17 ± 6.88, and 23.21 ± 5.22 points, respectively. Pearson correlation analysis showed that anxiety scores in the HADS were negatively correlated with CD-RISC score and SSRS score, with coefficients of –0.496 and –0.584, respectively (*p* < 0.05). And depression scores in the HADS were also negatively correlated with CD-RISC score and SSRS score, with coefficients of –0.496 and –0.482, respectively (*p* < 0.05). Multiple linear regression analysis showed that only CD-RISC and SSRS scores were independent predictors of postoperative anxiety and depression (*p* < 0.05).

**Conclusions:**

The levels of anxiety and depression were elevated in PTC patients after surgery. Both psychological resilience and social support were influencing factors for anxiety and depression in PTC patients.

## Introduction

The incidence of thyroid cancer has been on the rise over the past few decades, with papillary thyroid carcinoma (PTC) being the most common pathological type [1]. Currently, surgical intervention is the primary treatment for PTC, and adjuvant therapies such as endocrine therapy and radiotherapy, are administered postoperatively. The prognosis is generally favourable, but a small number of patients may experience recurrence or even death [2].

The increased psychological burden caused by surgical procedures and adjuvant treatments have substantially elevated the physical and mental stress on patients. A study indicated that PTC patients exhibit higher anxiety scores while awaiting pathological results [3]. Depression and anxiety are the most common adverse emotional symptoms in patients after PTC surgery. Previous studies have shown that PTC patients typically exhibit higher levels of anxiety and depression compared with healthy populations [4, 5]. Psychological resilience refers to the adaptive process of quickly recovering and maintaining mental health when facing heavy stress [6]. A study has shown that psychological resilience is negatively correlated with postoperative anxiety and depression in thyroid cancer patients [7], indicating that psychological resilience is an important factor affecting the mental health of thyroid cancer patients. Additionally, research indicates that insufficient social support can exacerbate feelings of loneliness [8, 9], while both inadequate social support and loneliness increase the risk of postoperative depression and anxiety [10].

However, studies on the correlation between the anxiety and depression status of PTC patients and the aforementioned factors are limited. Therefore, this study aimed to investigate the status of anxiety and depression in postoperative patients with PTC using questionnaires, and to analyse their associations with psychological resilience and social support.

## Materials and Methods

### Study Subjects

A total of 122 patients with PTC who underwent surgical treatment at our hospital between January 2022 and June 2023 were enrolled using a continuous recruitment method. Inclusion criteria: (1) postoperative pathological results confirmation of PTC [11]; (2) having undergone thyroid lobectomy or total thyroidectomy; (3) at least 1 month since discharge (the mean time from surgery to assessment was 45.2 ± 10.3 days, ranging from 32 to 68 days); and (4) provision of informed consent by patients and their family members. Exclusion criteria: (1) presence of other severe primary diseases or malignant tumours; (2) incomplete clinical data; and (3) coexisting mental disorders or cognitive impairment, making it difficult to understand the study content and cooperate with the questionnaire survey.

This study was approved by the Medical Ethics Committee of Hefei First People’s Hospital (PJ-2021-L068). This study was conducted in full accordance with the ethical principles of the Declaration of Helsinki and its subsequent amendments [12].

### Study Methods

Clinical data of patients during hospitalisation were collected, including medical payment, comorbid chronic diseases, ^131^I therapy, surgical approach, and lymph node dissection. Data were collected via face-to-face questionnaire surveys.

At the one-month postoperative follow-up visits, trained researchers provided patients and their family members with detailed explanations regarding the study objectives, questionnaire instructions, and confidentiality principles. Following the acquisition of written informed consent, questionnaires were distributed and completed on-site. During completion, researchers remained present to offer one-on-one guidance and standardised responses to patient inquiries, while strictly avoiding leading instructions, ensuring that patients fully understood each item and complete the questionnaire independently. Upon completion, researchers immediately collected the questionnaires and conducted on-site reviews to verify completeness (checking for missing or incorrectly filled items). For items containing logical inconsistencies or non-standard entries, researchers promptly verified and corrected them with the patients to ensure validity.

After collection, data were entered into an Excel database using a double-entered method by two independent researchers. Cross-validation was performed upon completion of the entry, and any discrepancies identified were traced back to the original source for correction, thereby guaranteeing data accuracy.

The questionnaires included baseline demographic data (age, gender, educational level, marital status, number of children, employment status, and income level), the Connor–Davidson Resilience Scale (CD-RISC) [13], the Social Support Rating Scale (SSRS) [14], and the Hospital Anxiety and Depression Scale (HADS) [15].

(1) CD-RISC: This scale consists of 25 items across three dimensions (tenacity, strength, and optimism). Each item is scored on a 5-point Likert scale (0–4 points). The total scores for the three dimensions are 52, 32, and 16 points, respectively, with an overall score of 100 points. Higher scores indicate better psychological resilience (Cronbach’s α = 0.89) [13].

(2) SSRS: This scale includes 10 items across three dimensions (objective support, subjective support, and support utilisation). The total scores for the three dimensions are 32, 22, and 12 points, respectively. The overall scale score is weighted by the scores of the three dimensions, ranging from 12 to 66 points [16]. Higher scores indicate higher levels of social support (Cronbach’s α for each item: 0.89–0.94) [14].

(3) HADS: The HADS consists of two subscales (anxiety and depression) with a total of 14 items. Each item is scored on a 4-point scale (0–3). Each subscale has a total score of 21 points, resulting in an overall total of 42 points. Higher scores indicate more severe symptoms of anxiety and depression (Cronbach’s α = 0.85). Scores of 0-7 indicate mild anxiety or depression, 8-14 indicate moderate anxiety or depression, and 15–21 indicate severe anxiety or depression, which was adopted to maintain consistency with the referenced study [17].

### Statistical Methods

Data entry and calculation were performed using Excel (Microsoft Corp., Redmond, WA, USA) software, and statistical analysis was conducted with SPSS 24.0 software (IBM Corp., Armonk, NY, USA). The normality of continuous variables was assessed using the Kolmogorov-Smirnov test. A *p*-value > 0.05 indicated a normal distribution. Normally distributed data were presented as mean ± standard deviation (SD) and compared using the independent-sample *t*-test or one-way Analysis of Variance (ANOVA). Non-normally distributed data were expressed as median (interquartile range, IQR). Categorical data were presented as number (percentage) and compared using the chi-square test. Pearson correlation analysis was applied to analyse the correlations between HADS, CD-RISC, and SSRS scores. Multiple linear regression analysis was used to identify the influencing factors of anxiety and depression in PTC patients after surgery. A two-tailed *p* value < 0.05 was considered statistically significant.

## Results

### Baseline Demographic Data of PTC Patients

All questionnaires were effectively collected. The majority of participants were aged 40–59 years (46.7%), were predominantly female (82.8%), and were mostly married (77.1%) with children (66.4%). Most participants were covered by health insurance (86.9%), and the majority did not undergo lymph node dissection (62.3%). Other baseline characteristics were relatively well-balanced across the groups (Table 1).

**Table 1.** **Baseline demographic data and clinical characteristics of PTC patients**.

Variables		n (%)
Age (years)		
	<40	40 (32.8)
	40–59	57 (46.7)
	>60	25 (20.5)
Gender		
	Male	21 (17.2)
	Female	101 (82.8)
Educational level		
	Junior high school or below	33 (27.1)
	High school or junior college	47 (38.5)
	Bachelor’s degree or above	42 (34.4)
Marital status		
	Married	94 (77.1)
	Unmarried	28 (22.9)
Number of children		
	With children	81 (66.4)
	Without children	41 (33.6)
Monthly income (RMB)		
	<3000	29 (23.8)
	3000–5000	55 (45.1)
	>5000	38 (31.1)
Employment status		
	Employed	61 (50.0)
	Retired	46 (37.7)
	Unemployed	15 (12.3)
Payment method		
	Self-paid	16 (13.1)
	Insurance	106 (86.9)
Comorbid chronic diseases		
	Yes	51 (41.8)
	No	71 (58.2)
^131^I therapy		
	Yes	64 (52.5)
	No	58 (47.5)
Surgical approach		
	Thyroid Lobectomy	50 (41.0)
	Total Thyroidectomy	72 (59.0)
Lymph node dissection		
	Yes	46 (37.7)
	No	76 (62.3)

PTC, papillary thyroid carcinoma. At the time of data collection (January 2022 to June 2023), the average exchange rate was approximately 1 USD = 6.85 RMB (range: 6.70–6.95). Thus, the monthly income brackets of <3,000 RMB, 3,000–5,000 RMB, and >5,000 RMB correspond approximately to <440 USD, 440–730 USD, and >730 USD, respectively.

### CD-RISC Scores of PTC Patients After Surgery

The total CD-RISC score of 122 PTC patients after surgery was 80.00 ± 7.41 points. The scores of each dimension are shown in Table 2.

**Table 2.** **CD-RISC score of PTC patients after surgery ($\bar{x}$ ± s, points)**.

Dimension	n	Score
Tenacity	122	43.14 ± 5.38
Strength	122	25.02 ± 4.10
Optimism	122	11.80 ± 3.07
Total score	122	80.00 ± 7.41

CD-RISC, Connor-Davidson Resilience Scale; PTC, papillary thyroid carcinoma.

### SSRS Scores of PTC Patients After Surgery

The total SSRS score of 122 PTC patients after surgery was 53.17 ± 6.88 points. The scores of each dimension are shown in Table 3.

**Table 3.** **SSRS scores of PTC patients after surgery ($\bar{x}$ ± s, points)**.

Dimension	n	Score
Objective support	122	17.44 ± 2.84
Subjective support	122	26.16 ± 4.05
Support utilisation	122	9.13 ± 2.09
Total score	122	53.17 ± 6.88

SSRS, Social Support Rating Scale; PTC, papillary thyroid carcinoma.

### HADS Scores of PTC Patients After Surgery

The total HADS score of 122 PTC patients after surgery was 23.21 ± 5.22 points. The scores of each dimension and the levels of anxiety and depression are shown in Table 4.

**Table 4.** **HADS scores of PTC patients after surgery**.

Category		n	Score/Percentage
HADS scores ($\bar{x}$ ± s, points)			
	Anxiety	122	11.63 ± 3.07
	Depression	122	11.53 ± 3.21
	Total score	122	23.21 ± 5.22
Anxiety level (%)			
	Mild anxiety	12	9.9
	Moderate anxiety	94	77.0
	Severe anxiety	16	13.1
Depression level (%)			
	Mild depression	9	7.4
	Moderate depression	94	77.0
	Severe depression	19	15.6

HADS, Hospital Anxiety and Depression Scale; PTC, papillary thyroid carcinoma.

### Correlation Analysis of Psychological Resilience, Social Support and Anxiety and Depression in PTC Patients After Surgery

**Fig. 1.**
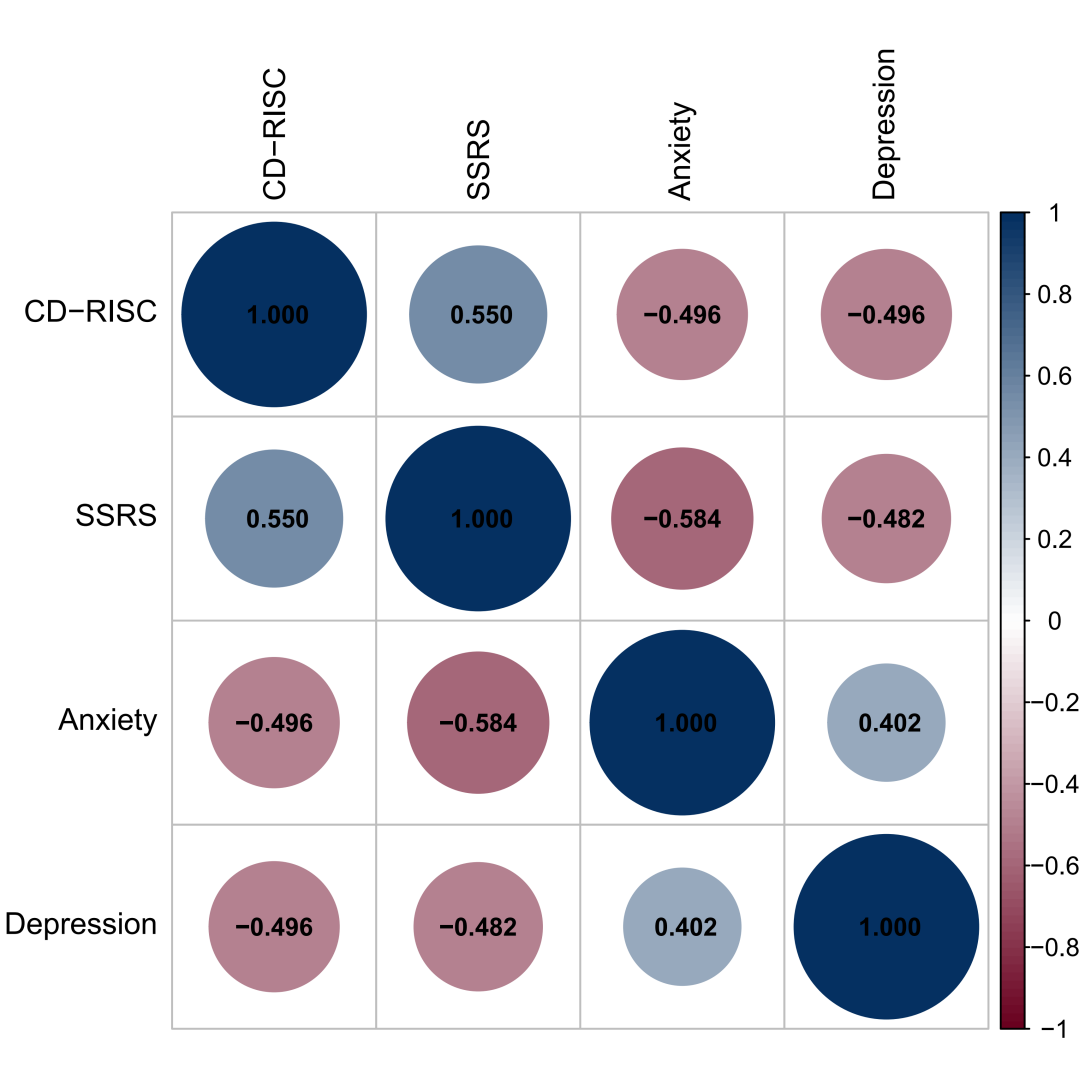
**Correlation coefficient plot of CD-RISC scores, SSRS scores and HADS scores**. The size of the circle is positively correlated with the correlation coefficient. Red indicates a positive correlation, and blue indicates a negative correlation. CD-RISC, Connor-Davidson Resilience Scale; SSRS, Social Support Rating Scale; HADS, Hospital Anxiety and Depression Scale.

Pearson correlation analysis showed that anxiety score was negatively correlated with the CD-RISC score and SSRS score, with correlation coefficients of –0.496 and –0.584, respectively (*p* < 0.05) (Table 5, Fig. 1). The depression score of PTC patients after surgery was negatively correlated with the CD–RISC score and SSRS score, with correlation coefficients of –0.496 and –0.482, respectively (*p* < 0.05) (Table 5, Fig. 1).

**Table 5.** **Pearson correlation coefficient of CD-RISC, SSRS and HADS scores**.

Scales	CD–RISC		SSRS		Anxiety		Depression	
	r	*p*	r	*p*	r	*p*	r	*p*
CD–RISC	1.000	0.000	0.550	<0.001	–0.496	<0.001	–0.496	<0.001
SSRS	0.550	<0.001	1.000	0.000	–0.584	<0.001	–0.482	<0.001
Anxiety	–0.496	<0.001	–0.584	<0.001	1.000	0.000	0.402	<0.001
Depression	–0.496	<0.001	–0.482	<0.001	0.402	<0.001	1.000	0.000

CD-RISC, Connor-Davidson Resilience Scale; SSRS, Social Support Rating Scale; HADS, Hospital Anxiety and Depression Scale.

### Linear Regression Analysis of the Effects of Psychological Resilience and Social Support on Anxiety in PTC Patients After Surgery

Univariate linear regression analysis indicated that patients with lower monthly income, comorbid chronic diseases, and self-paid treatment had significantly higher anxiety scores (*p* < 0.1) (Table 6). Patients with higher CD-RISC and SSRS scores had significantly lower anxiety scores (*p* < 0.1) (Table 6).

**Table 6.** **Univariate linear regression analysis of anxiety in PTC patients after surgery**.

Variables		β	S.E.	*t*	*p*	β (95% *CI*)	VIF
Age (years)							1.069
	<40					Ref	
	40–59	–0.09	0.64	–0.15	0.883	–0.09 (–1.34–1.15)	
	>60	0.61	0.79	0.78	0.439	0.61 (–0.93–2.15)	
Gender							1.136
	Male					Ref	
	Female	0.93	0.73	1.27	0.205	0.93 (–0.50–2.37)	
Educational level							1.138
	Junior high school or below					Ref	
	High school or junior college	–0.56	0.65	–0.85	0.397	–0.56 (–1.84–0.73)	
	Bachelor’s degree or above	–0.66	0.72	–0.92	0.359	–0.66 (–2.07–0.74)	
Marital status							1.052
	Married					Ref	
	Unmarried	–0.36	0.66	–0.54	0.593	–0.36 (–1.65–0.94)	
Number of children							1.099
	With children					Ref	
	Without children	0.34	0.59	0.57	0.571	0.34 (–0.82–1.49)	
Monthly income (RMB)							1.131
	<3000					Ref	
	3000–5000	–0.71	0.70	–1.01	0.315	–0.71 (–2.08–0.67)	
	>5000	–1.27	0.75	–1.68	**0.096**	–1.27 (–2.74–0.21)	
Employment status							1.129
	Employed					Ref	
	Retired	0.09	0.60	0.15	0.883	0.09 (–1.09–1.27)	
	Unemployed	–0.61	0.89	–0.68	0.498	–0.61 (–2.35–1.14)	
Payment method							1.187
	Self-paid					Ref	
	Insurance	–1.72	0.81	–2.12	**0.036**	–1.72 (–3.31– –0.13)	
Comorbid chronic diseases							1.163
	Yes					Ref	
	No	–0.97	0.56	–1.74	**0.085**	–0.97 (–2.07–0.12)	
^131^I therapy							1.251
	Yes					Ref	
	No	–0.45	0.56	–0.8	0.424	–0.45 (–1.54–0.65)	
Surgical approach							1.157
	Thyroid Lobectomy					Ref	
	Total Thyroidectomy	0.46	0.57	0.81	0.419	0.46 (–0.65–1.57)	
Lymph node dissection							1.150
	Yes					Ref	
	No	–0.70	0.57	–1.22	0.226	–0.70 (–1.82–0.43)	
CD-RISC (scores)		–0.21	0.03	–6.27	< **0.001**	–0.21 (–0.27– –0.14)	1.585
SSRS (scores)		–0.26	0.03	–7.89	< **0.001**	–0.26 (–0.33– –0.20)	1.683

CD-RISC, Connor-Davidson Resilience Scale; CI, confidence interval; SSRS, Social Support Rating Scale; PTC, papillary thyroid carcinoma; S.E., standard error; VIF, Variance Inflation Factor. At the time of data collection (January 2022 to June 2023), the average exchange rate was approximately 1 USD = 6.85 RMB (range: 6.70–6.95). Thus, the monthly income brackets of <3,000 RMB, 3,000–5,000 RMB, and >5,000 RMB correspond approximately to <440 USD, 440–730 USD, and >730 USD, respectively. Bold fonts indicate *p* < 0.1.

**Fig. 2.**
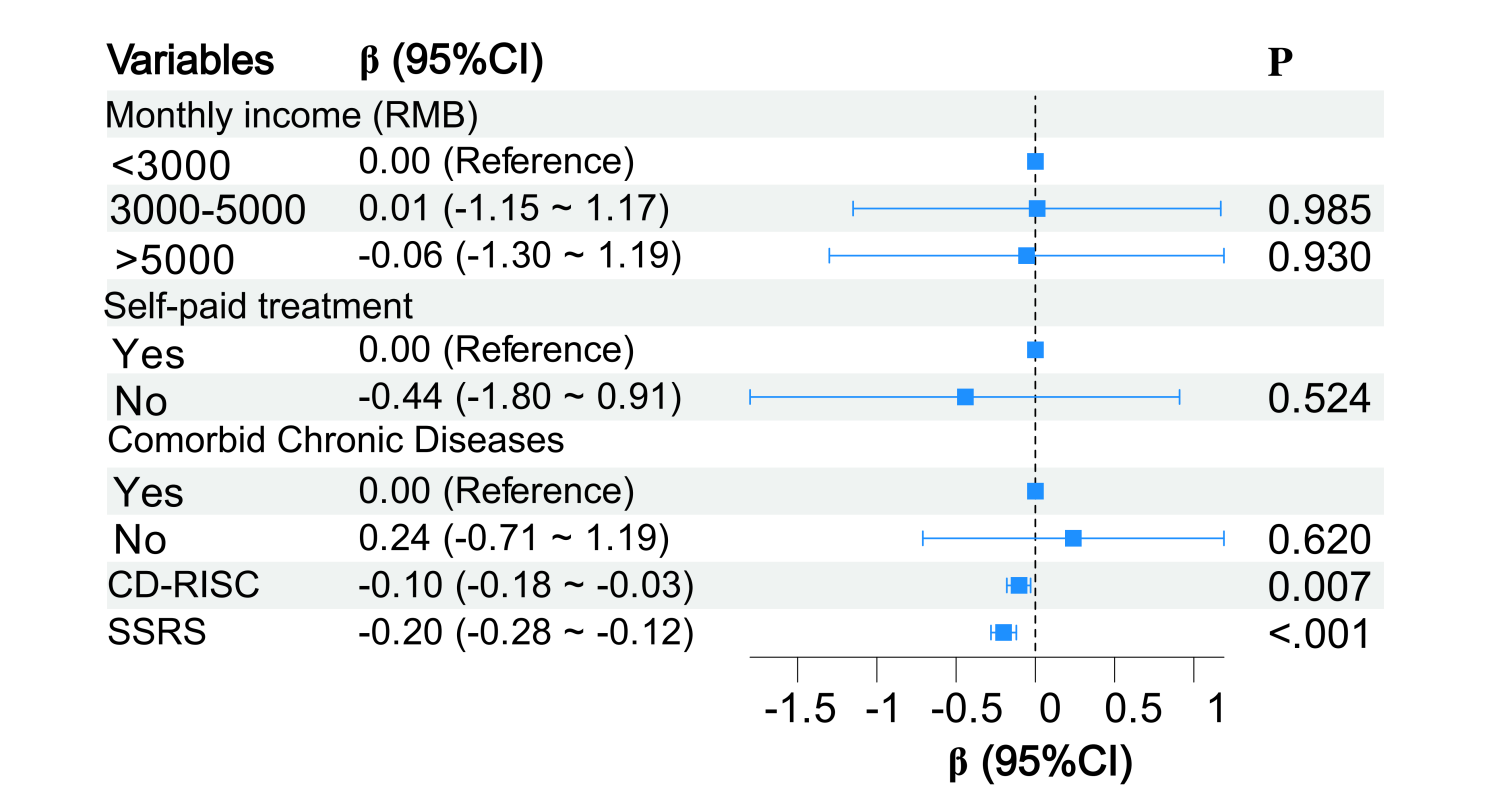
**Forest plot of multivariate analysis for postoperative anxiety**. CD-RISC, Connor-Davidson Resilience Scale; SSRS, Social Support Rating Scale; CI, confidence interval.

Variables identified in the univariate analysis were entered into a multiple linear regression model, with anxiety scores as dependent variables. The results showed that only CD-RISC and SSRS scores were independent predictors for anxiety in patients with PTC (*p* < 0.05) (Fig. 2). The model demonstrated an R^2^ of 0.299, an adjusted R^2^ of 0.207, and an overall F-statistic of 3.262.

To avoid omitting confounding variables with established clinical significance despite non-significance in univariate analysis (e.g., age), we constructed a supplementary model. This multivariate analysis included age, sex, monthly income, and the extent of lymph node dissection. After adjusting for these confounders, both CD-RISC and SSRS remained independent predictors of anxiety (**Supplementary Table 1**).

### Linear Regression Analysis of the Effects of Psychological Resilience and Social Support on Depression in PTC Patients After Surgery

Univariate linear regression analysis results indicated that patients with lower monthly income, comorbid chronic diseases, ^131^I Therapy, and lymph node dissection and education level had significantly higher depression scores (*p* < 0.1) (Table 7). Patients with higher CD-RISC and SSRS scores had significantly lower depression scores (*p* < 0.1) (Table 7).

**Table 7.** **Univariate linear regression analysis of depression in PTC patients after surgery**.

Variables			β	S.E.	*t*	*p*	β (95% *CI*)	VIF
Age (years)								1.069
	<40						0.00 (Reference)	
	40–59		–0.19	0.67	–0.28	0.780	–0.19 (–1.49–1.12)	
	>60		–0.03	0.82	–0.03	0.976	–0.03 (–1.64–1.59)	
Gender								1.134
	Male						0.00 (Reference)	
	Female		0.70	0.77	0.91	0.364	0.70 (–0.81–2.21)	
Educational level								1.138
	Junior high school or below						0.00 (Reference)	
	High school or junior college		–1.19	0.68	–1.76	**0.081**	–1.19 (–2.52–0.14)	
	Bachelor’s degree or above		–0.82	0.74	–1.11	0.271	–0.82 (–2.28–0.63)	
Marital status								1.052
	Married						0.00 (Reference)	
	Unmarried		0.10	0.69	0.14	0.890	0.10 (–1.26–1.46)	
Number of children								1.099
	With children						0.00 (Reference)	
	Without children		0.23	0.62	0.37	0.715	0.23 (–0.98–1.44)	
Monthly income (RMB)								1.131
	<3000						0.00 (Reference)	
	3000–5000		–0.64	0.73	–0.87	0.383	–0.64 (–2.08–0.80)	
	>5000		–1.35	0.79	–1.71	**0.090**	–1.35 (–2.89–0.20)	
Employment status								1.129
	Employed						0.00 (Reference)	
	Retired		0.27	0.63	0.43	0.670	0.27 (–0.97–1.51)	
	Unemployed		0.04	0.93	0.04	0.965	0.04 (–1.78–1.87)	
Payment method								1.187
	Self-paid						0.00 (Reference)	
	Insurance		–1.40	0.85	–1.64	0.104	–1.40 (–3.08–0.27)	
Comorbid chronic diseases								1.163
	Yes						0.00 (Reference)	
	No		–1.71	0.57	–3.00	**0.003**	–1.71 (–2.83– –0.60)	
¹³¹I therapy								1.251
	Yes						0.00 (Reference)	
	No		–1.67	0.56	–2.97	**0.004**	–1.67 (–2.78– –0.57)	
Surgical approach								1.157
	Thyroid Lobectomy						0.00 (Reference)	
Total Thyroidectomy			0.84	0.59	1.42	0.158	0.84 (–0.32–1.99)	
	Lymph Node Dissection							1.150
		Yes					0.00 (Reference)	
		No	–1.45	0.59	–2.47	**0.015**	–1.45 (–2.60– –0.30)	
CD–RISC (scores)			–0.21	0.03	–6.25	< **0.001**	–0.21 (–0.28– –0.15)	1.585
SSRS (scores)			–0.22	0.04	–6.03	< **0.001**	–0.22 (–0.30– –0.15)	1.683

CD-RISC, Connor-Davidson Resilience Scale; CI, confidence interval; SSRS, Social Support Rating Scale; PTC, papillary thyroid carcinoma; S.E, standard error; VIF, Variance Inflation Factor. At the time of data collection (January 2022 to June 2023), the average exchange rate was approximately 1 USD = 6.85 RMB (range: 6.70–6.95). Thus, the monthly income brackets of <3,000 RMB, 3,000–5,000 RMB, and >5,000 RMB correspond approximately to <440 USD, 440–730 USD, and >730 USD, respectively. Bold fonts indicate *p* < 0.1.

**Fig. 3.**
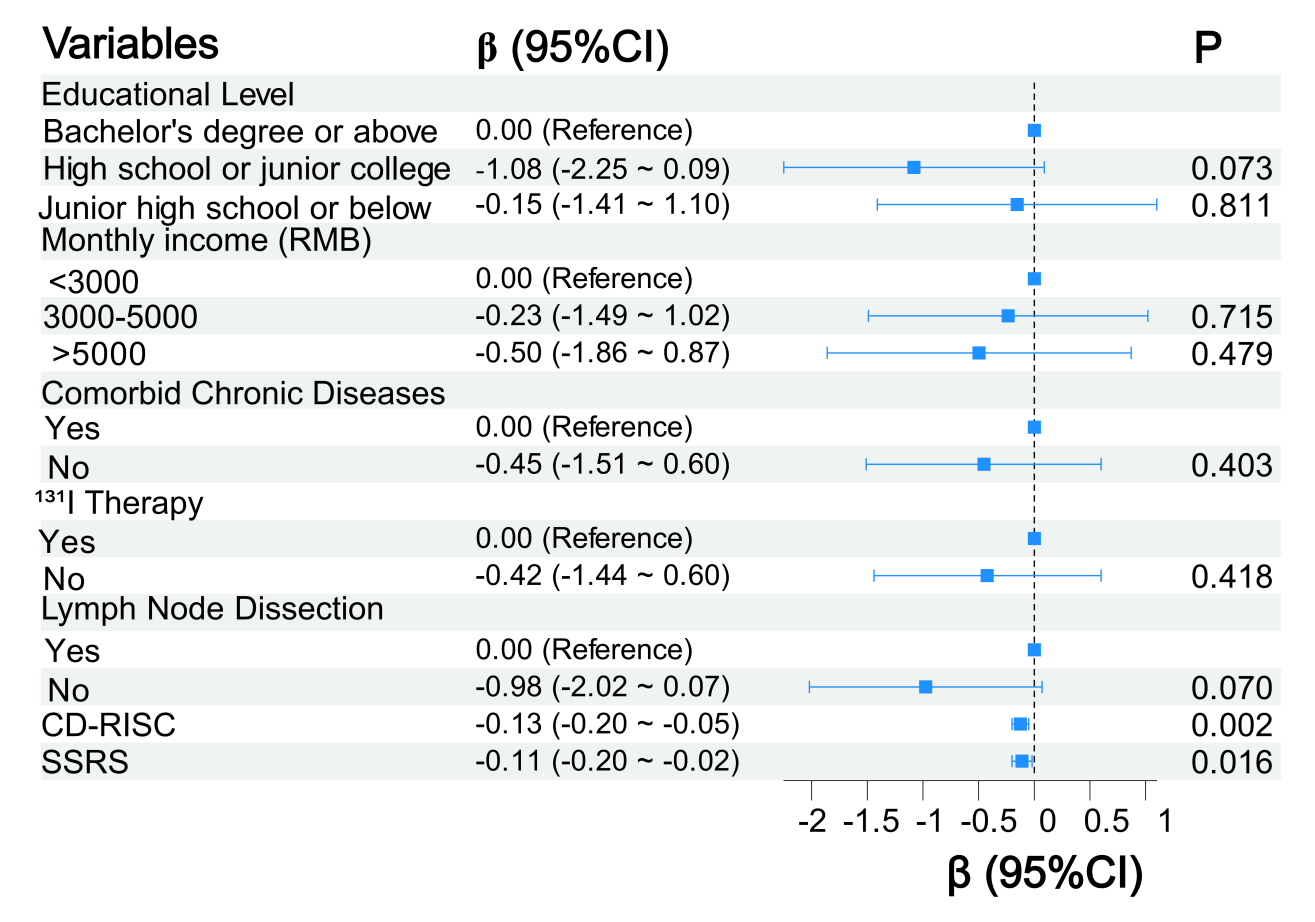
**Forest plot of multivariate analysis for postoperative depression**. CD-RISC, Connor-Davidson Resilience Scale; CI, confidence interval; SSRS, Social Support Rating Scale.

Variables identified in the univariate analysis were entered into a multiple linear regression model, with depression scores as dependent variables. The results showed that only CD-RISC and SSRS scores were independent predictors for depression in patients with PTC (*p* < 0.05) (Fig. 3). The model demonstrated an R^2^ of 0.367, an adjusted R^2^ of 0.284, and an overall F-statistic of 4.436.

To avoid omitting confounding variables with established clinical significance despite non-significance in univariate analysis (e.g., age), we constructed a supplementary model. This multivariate analysis included age, sex, monthly income, and the extent of lymph node dissection. After adjusting for these confounders, both CD-RISC and SSRS remained independent predictors of depression (see **Supplementary Table 2**).

## Discussion

Thyroid cancer is the most common tumour of the endocrine system, with PTC representing the predominant histological subtype. Epidemiological statistics show that in 2022, there were 466,100 new cases of thyroid cancer in China, ranking the third among all malignant tumors, following lung cancer and colorectal cancer [18]. Anxiety is a common mental disorder that not only impairs of physiological functioning but also increases the economic burden on individuals and society [19]. Multiple endocrine diseases have been shown to be associated with anxiety [20]. Previous studies have indicated that depression and anxiety are common among cancer patients, with reported rates of up to 37% [21]. In patients with thyroid cancer, anxiety is a key factor that seriously impair quality of life [5, 22]. Patients with PTC have a relatively short postoperative hospital stay and generally favourable prognosis, with a 10-year survival rate exceeding 95% [23]. However, factors such as tumour capsule invasion or lymph node metastasis may still lead to poor prognosis [24]. In the present study, the HADS scale was used to assess anxiety and depression symptoms. The mean HADS score of 122 patients with PTC in this study were 23.21 ± 5.22, and the majority of patients (77.0% for both anxiety and depression) exhibited moderate levels of symptoms.

Psychological resilience is a key determinant of mental health. Individuals with higher levels of resilience exhibit greater psychological endurance and adaptability to adversity. Studies have shown that good psychological resilience is conducive to the mental health and postoperative rehabilitation of patients with thyroid cancer [25]. In the present study, the CD-RISC scale was used to evaluate psychological resilience, yielding a total mean score of 80.00 ± 7.41 points among 122 PTC patients after surgery. Compared with a recent study, in which the CD-RISC score of postoperative thyroid cancer patients was 55.15 ± 6.28 points, the level of psychological resilience was higher in our study [26]. We hypothesise that this favourable resilience may be attributed to several factors. First, PTC typically progresses slowly, and even when lymph node metastases are present, they are often localised and associated with a favourable prognosis. Additionally, advancements in minimally invasive surgical techniques have reduced the risk of postoperative complications in recent years. Furthermore, preoperative and postoperative health education enables PTC patients to fully understand the disease progression and prognosis, thereby alleviating psychological burden and enhancing resilience.

Adequate social support promotes patient recovery and improves physical and mental health. Previous studies have indicated that most of the social support for patients after thyroid surgery comes from the family, mainly from their spouses [27]. In the present study, the SSRS was used to assess social support, with a mean total score of 53.17 ± 6.88 points among 122 PTC patients after surgery. In contrast, Li *et al*. [28] reported a significantly lower mean SSRS score of 38.41 points in thyroid cancer survivors, which may be attributed to the favourable prognosis and high survival rate of thyroid cancer, which may lead to relatively limited community and family support.

Multiple linear regression analysis in our study revealed that only CD-RISC and SSRS scores were independent predictors of symptoms of the anxiety and depression. The higher the psychological resilience and social support, the milder the symptoms of anxiety and depression. Individuals with lower psychological resilience, due to their insufficient ability to adapt to stressful life events, are more likely to experience adverse mental health outcomes, including depression and anxiety [29]. PTC patients with strong psychological resilience can adopt a positive attitude toward treatment, effectively adjust themselves and cope with the disease with positive emotions. This, in turn, helps to maintain mental and psychological health, reduce the negative impacts caused by stress, and thus reduce the risk of anxiety and depression [30]. Our findings align with previous studies, which have demonstrated that “adapting to change” is negatively correlated with mental health disorders such as anxiety and depression [31, 32]. Existing studies have shown that numerous approaches may alleviate anxiety, among which social support has emerged as a key focus of relevant research [33]. The main effect hypothesis of social support holds that social support exerts a positive impact regardless of an individual’s stress level [34]. One study found that women with lower social support experienced more severe anxiety symptoms than those with stable family backgrounds. Patients who were married or in stable partnerships had significantly lower anxiety and depression scores than those who were divorced or living alone at the same time point [35]. These findings indicate that low social support is an independent predictor of increased anxiety and depression. The medial prefrontal cortex–amygdala pathway may play a key role in this process, as it has been identified as a core mechanism underlying the reduction of anxiety and the enhancement of social support by means of top-down regulation of emotional responses [36].

This study has certain limitations that need to be acknowledged and addressed in future research. First, the sample size involved in this study was relatively small, which may compromise the statistical power of the analysis and limited the generalisability of the findings. Second, the observation period was relatively short. Short-term follow-up and observation may fail to capture the long-term dynamic changes in anxiety and depression symptoms of PTC patients. Moreover, due to the lack of comprehensive baseline data, low incidence of heterogeneity in tumour staging and postoperative complications, potential factors such as preoperative psychological status, tumour staging (TNM staging), and postoperative complications were not directly included in our study. Future studies with larger sample sizes and longer follow-up periods are therefore warranted to provide more robust and comprehensive evidence.

## Conclusions

PTC patients exhibited low levels of anxiety and depression. Psychological resilience and social support are independent predictors of both anxiety and depression symptoms. Therefore, it is recommended that PTC patients strengthen the development of psychological resilience training (e.g., cognitive behavioural therapy, mindfulness-based stress reduction) and establish a multi-dimensional social support network (e.g., encompassing medical support, family support, and peer mutual assistance) during the postoperative recovery period.

## Availability of Data and Materials

All experimental data included in this study can be obtained by contacting the corresponding author if needed.
