# Supplementary material for: Effects of Psychological Resilience and Social Support on Anxiety and Depression in Patients With Papillary Thyroid Carcinoma
Source: Actas Esp Psiquiatr. 2026 Apr 15;54(2):432–42. doi: 10.62641/aep.v54i2.2181 (PMC13180683; doi:10.62641/aep.v54i2.2181)
Supplement: Supplementary file 1 [file ActEsp-54-2-432-442-s1.zip › Supplementary Tables.docx]

Supplementary Tables

Supplementary Table 1 Multivariate linear regression analysis of anxiety in PTC patients after inclusion of additional confounding factors

| Variables | β | S.E | t | *P* | β (95% CI) |
| --- | --- | --- | --- | --- | --- |
| Age (years) |  |  |  |  |  |
| <40 |  |  |  |  | Ref |
| 40-59 | -0.53 | 0.52 | -1.01 | 0.313 | -0.53 (-1.55 ~ 0.49) |
| >60 | 0.25 | 0.63 | 0.4 | 0.693 | 0.25 (-0.98 ~ 1.48) |
| Gender |  |  |  |  |  |
| Male |  |  |  |  | Ref |
| Female | 0.79 | 0.59 | 1.34 | 0.184 | 0.79 (-0.37 ~ 1.95) |
| Monthly income (RMB) |  |  |  |  |  |
| <3000 |  |  |  |  | Ref |
| 3000-5000 | -0.1 | 0.6 | -0.16 | 0.872 | -0.10 (-1.27 ~ 1.08) |
| >5000 | -0.16 | 0.65 | -0.25 | 0.805 | -0.16 (-1.43 ~ 1.11) |
| Lymph Node Dissection |  |  |  |  |  |
| Yes |  |  |  |  | Ref |
| No | 0 | 0.47 | 0.01 | 0.995 | 0.00 (-0.93 ~ 0.93) |
| CD-RISC | -0.1 | 0.04 | -2.87 | **0.005** | -0.10 (-0.18 ~ -0.03) |
| SSRS | -0.2 | 0.04 | -4.9 | **<.001** | -0.20 (-0.28 ~ -0.12) |

CD-RISC: Connor-Davidson Resilience Scale; CI: confidence interval; SSRS: Social Support Rating Scale; PTC: papillary thyroid carcinoma; S.E: standard error;

Supplementary Table 2 Multivariate linear regression analysis of depression in PTC patients after inclusion of additional confounding factors

| Variables | β | S.E | t | *P* | β (95% *CI*) |
| --- | --- | --- | --- | --- | --- |
| Age (years) |  |  |  |  |  |
| <40 |  |  |  |  | Ref |
| 40-59 | -0.66 | 0.58 | -1.14 | 0.256 | -0.66 (-1.79 ~ 0.47) |
| >60 | -0.28 | 0.69 | -0.4 | 0.688 | -0.28 (-1.64 ~ 1.08) |
| Gender |  |  |  |  |  |
| Male |  |  |  |  | Ref |
| Female | 0.61 | 0.65 | 0.93 | 0.352 | 0.61 (-0.67 ~ 1.89) |
| Monthly income (RMB) |  |  |  |  |  |
| <3000 |  |  |  |  | Ref |
| 3000-5000 | -0.29 | 0.66 | -0.44 | 0.659 | -0.29 (-1.59 ~ 1.00) |
| >5000 | -0.6 | 0.72 | -0.84 | 0.403 | -0.60 (-2.01 ~ 0.80) |
| Lymph Node Dissection |  |  |  |  |  |
| Yes |  |  |  |  | Ref |
| No | -0.83 | 0.52 | -1.59 | 0.115 | -0.83 (-1.86 ~ 0.19) |
| CD-RISC | -0.14 | 0.04 | -3.38 | **<.001** | -0.14 (-0.21 ~ -0.06) |
| SSRS | -0.13 | 0.04 | -2.88 | **0.005** | -0.13 (-0.22 ~ -0.04) |

CD-RISC: Connor-Davidson Resilience Scale; CI: confidence interval; SSRS: Social Support Rating Scale; PTC: papillary thyroid carcinoma; S.E: standard error;
